# Supplementary material for: Plant diversity effects on forage quality, yield and revenues of semi-natural grasslands
Source: Nat Commun. 2020 Feb 7;11:768. doi: 10.1038/s41467-020-14541-4 (PMC7005841; doi:10.1038/s41467-020-14541-4)
Supplement: Supplementary file 1 — Supplementary Information [file 41467_2020_14541_MOESM1_ESM.pdf]

Supplementary Information

Supplementary Tables

Supplementary Table 1: Management intensities. Management intensities were established on subplots within larger experimental plots except the less intensive management that represents the management intensity of the large plots. Cutting frequency is given in cuts per year, all fertilization levels are given in kg ha^{-1} a^{-1}. Nitrogen was applied as NO_3-N and NH_4-N in equal proportions, phosphorus as P_2O_5-P and Potassium as K_2O-K.

Management intensity	Cutting frequency	Fertilization		
		N	P	K
Extensive	1	0	0	0
Less intensive	2	0	0	0
Intensive	2	100	43.6	83
Highly intensive	4	100	43.6	83
Very highly intensive	4	200	87.2	166

Supplementary Table 2: Variable description: biomass yield, forage quality and revenues. The respective quality-adjusted yield variables are calculated by multiplying the below mentioned variables with biomass yield.

Name	Description
Biomass yield (g m^{-2})	Biomass is the dry weight of plants of an area at a given time.
Organic matter content (g kg^{-1})	Organic matter is the part of biomass that is not ash.
Neutral detergent fiber content (g kg^{-1})	Neutral detergent fiber describes the total fiber content in forage.
Crude protein content (g kg^{-1})	Crude protein includes true protein and other N-containing compounds and is calculated by convention as crude protein = nitrogen × 6.25.
(Metabolically) Utilizable crude protein content (g kg^{-1})	Utilizable crude protein reflects the crude protein from microbial synthesis plus the ruminally undegradable protein which is available in the duodenum of ruminants.
Metabolizable energy content (MJ kg^{-1})	Metabolizable energy is the energy available in the metabolism of the animal to gain body weight or to produce milk.
Net energy of lactation (MJ kg^{-1})	Net energy of lactation represents the energy available for lactation (Net energy of lactation = metabolizable energy content – heat from digestion and metabolism).
Milk production potential (kg kg^{-1})	Milk production potential describes the amount of milk which theoretically can produced from the respective forage. It is based on optimal production condition and a cow with 630 kg of live-weight and an annual milk yield of 7000 kg.
Revenues (Euro ha^{-1})	Revenues = Milk production potential yield × milk price

Source: Ball et al. (1), Barnes et al. (2), Edmunds et al. (3) and Jans et al. (4).

Mixed Effect Model Results, Model with Legume Presence

Supplementary Table 3: Result of mixed effect model for annual average metabolizable energy content (MJ kg^{-1}), annual metabolizable energy yield (MJ m^{-2} a^{-1}), annual average milk production potential (kg kg^{-1} a^{-1}), annual milk production potential yield (kg m^{-2} a^{-1}). *, **, *** denote significance at the 5%, 1% and 0.1% level, respectively (corrected for multiple comparisons). Standard errors are corrected for heteroscedasticity. Numbers in parentheses are z-values, which are based on robust standard errors.

	Metabolizable energy content	Metabolizable energy yield	Milk production potential	Milk production potential yield
Constant	8.15 (27.45)***	0.32 (1.29)	1.49 (23.93)***	0.06 (1.47)
Plant diversity$^{0.5}$ ($D^{0.5}$)×Management (M)				
Extensive	-0.13 (-1.84)	0.68 (5.95)***	-0.03 (-1.95)	0.12 (5.9)***
Less intensive	-0.09 (-2)	0.81 (5.73)***	-0.02 (-2.01)	0.15 (5.69)***
Intensive	-0.12 (-2.73)*	1 (5.47)***	-0.03 (-2.73)*	0.18 (5.34)***
Highly intensive	0 (0.01)	0.66 (9.87)***	0 (-0.03)	0.13 (9.17)***
Very highly intensive	0.06 (1.42)	0.91 (4.73)***	0.01 (1.4)	0.18 (4.83)***
M				
Less intensive	0.62 (1.99)	1.21 (2.91)*	0.13 (2.04)	0.22 (2.99)*
Intensive	1 (3.58)**	3.08 (11.06)***	0.22 (3.63)**	0.57 (10.64)***
Highly intensive	2.08 (5.79)***	3.41 (24.14)***	0.46 (6.1)***	0.65 (21.31)***
Very highly intensive	2.34 (7.47)***	5.46 (54.88)***	0.52 (7.88)***	1.04 (66.86)***
Legumes (L)x M				
Extensive	0.48 (2.56)	1.52 (5.66)***	0.11 (2.61)	0.29 (5.86)***
Less intensive	0.76 (4.42)***	4.3 (7.13)***	0.17 (4.47)***	0.82 (7.25)***
Intensive	0.57 (5.43)***	2.25 (4.52)***	0.13 (5.75)***	0.44 (4.65)***
Highly intensive	0.52 (1.68)	2.32 (2.66)*	0.12 (1.66)	0.45 (2.73)*
Very highly intensive	0.16 (0.74)	0.73 (0.95)	0.04 (0.74)	0.14 (0.96)
Functional groups (FG)	-0.1 (-0.82)	-0.87 (-2.36)	-0.03 (-0.91)	-0.17 (-2.49)
Grasses (G)	0.11 (1.44)	1.78 (2.63)	0.03 (1.63)	0.34 (2.74)*
Tall Herbs (H)	-0.19 (-0.91)	0.21 (0.43)	-0.04 (-0.89)	0.04 (0.41)
Random effects {Variance components}				
Block (B)	0.004 {0.01}	<0.0001 {<0.0001}	0.0002 {0.0002}	<0.0001 {<0.0001}
Plot (P)	0.08 {0.02}	0.92 {9.04}	0.004 {0.0009}	0.03 {0.04}
Residual	0.43 {0.04}	3.64 {2.7}	0.02 {0.002}	0.13 {0.02}
Number of groups				
Block	4	4	4	4
Plot	79	79	79	79

Supplementary Table 4: Result of mixed effect model for annual average crude protein content (g kg^{-1}), annual crude protein yield (g m^{-2} a^{-1}), annual average utilizable crude protein content (g kg^{-1}) and annual utilizable crude protein yield (g m^{-2} a^{-1}). *, **, *** denote significance at the 5%, 1% and 0.1% level, respectively (corrected for multiple comparisons). Standard errors are corrected for heteroscedasticity. Numbers in parentheses are z-values, which are based on robust standard errors.

	Crude protein content	Crude protein yield	Utilizable crude protein content	Utilizable crude protein yield
Constant	121.29 (14.14)***	5.9 (1.59)	182.3 (25.52)***	22.07 (4.6)***
Plant diversity$^{0.5}$ ($D^{0.5}$)×Management (M)				
Extensive	-5.47 (-6.72)***	9.73 (6.72)***	-	-
Less intensive	-4.48 (-1.7)	10.45 (6.22)***	-4.16 (-1.8)	11.66 (10.63)***
Intensive	-2.59 (-6.46)***	14.77 (4.28)***	-2.55 (-1.46)	12.8 (6.14)***
Highly intensive	-0.63 (-0.31)	11.28 (6.7)***	-0.91 (-0.26)	5.87 (2.04)
Very highly intensive	0.78 (0.33)	17.26 (4.64)***	-0.59 (-0.19)	9.84 (3.96)***
M				
Less intensive	16.4 (2.08)	19.28 (4.02)***	-	-
Intensive	18.03 (5.6)***	38.94 (8.37)***	6.83 (0.91)	23.45 (4.25)***
Highly intensive	74.73 (9.11)***	65.9 (15.82)***	50.11 (6.28)***	-0.91 (-0.26)
Very highly intensive	96.24 (7.78)***	109.9 (12.22)***	63.77 (4.62)***	8.75 (1.96)
Legumes (L)xM				
Extensive	70.04 (18.77)***	35.78 (12.95)***	-	-
Less intensive	81.87 (16.33)***	101.22 (11.55)***	18.9 (2.91)*	79.47 (6.38)***
Intensive	63.64 (5.95)***	64.07 (4.75)***	-1.71 (-0.36)	45.77 (2.81)*
Highly intensive	52.45 (11.17)***	54.23 (5.3)***	2.78 (0.24)	36.16 (4.79)***
Very highly intensive	49.68 (5.07)***	32.99 (1.75)	-5.24 (-0.52)	29.65 (2.5)
Functional groups (FG)	-17.03 (-3.35)**	-19.5 (-3.32)**	12.67 (3.05)*	-15.15 (-4)***
Grasses (G)	-1.75 (-0.27)	25.19 (3.55)**	-39.28 (-5.27)***	18.73 (7.45)***
Tall Herbs (H)	5.76 (2.34)	8.31 (0.79)	-14.36 (-3.51)**	1.59 (0.21)
Random effects {Variance components}				
Block (B)	33.3 {35.84}	<0.0001 {<0.0001}	46.18 {40.24}	31.79 {28.21}
Plot (P)	258.94 {88.18}	341.81 {184.54}	114.34 {38.19}	340.74 {363.45}
Residual	480.46 {54.85}	1129.88 {190.35}	474.51 {93.55}	923.77 {127.71}
Number of groups				
Block	4	4	4	4
Plot	80	80	77	77

Note utilizable crude protein content was only measured for the first cut of the year.

Supplementary Table 5: Result of mixed effect model for annual average organic matter content (g kg^{-1}), annual organic matter yield (g m^{-2} a^{-1}), annual average neutral detergent fiber content (g kg^{-1}) and annual neutral detergent fiber yield (g m^{-2} a^{-1}). *, **, *** denote significance at the 5%, 1% and 0.1% level, respectively (corrected for multiple comparisons). Standard errors are corrected for heteroscedasticity. Numbers in parentheses are z-values, which are based on robust standard errors.

	Organic matter content	Organic matter yield	Neutral detergent fiber content	Neutral detergent fiber yield
Constant	896.51 (171.91)***	9.76 (0.4)	489.13 (23.16)***	-9.97 (-0.89)
Plant diversity$^{0.5}$ ($D^{0.5}$)×Management (M)				
Extensive	3.14 (5.04)***	85.93 (6.48)***	24.99 (5.29)***	64.71 (6.57)***
Less intensive	2.86 (2.74)*	92.22 (6.04)***	13.85 (2.82)*	65.54 (5.72)***
Intensive	2.5 (2.23)	117.66 (6.31)***	18.65 (5.2)***	85.12 (10.15)***
Highly intensive	1.66 (0.83)	66.78 (21.1)***	3.24 (0.92)	28.74 (10.53)***
Very highly intensive	0.71 (1.43)	73.08 (4.08)***	8.34 (2.34)	40.88 (4.06)***
M				
Less intensive	-0.43 (-0.07)	131.32 (2.75)*	-24.46 (-3.7)**	71.9 (2.1)
Intensive	0.89 (0.14)	308.88 (10.77)***	-28.58 (-1.5)	176.09 (5.81)***
Highly intensive	-17.58 (-2.61)	277.17 (12.56)***	-136.34 (-6.88)***	138.31 (10.93)***
Very highly intensive	-10.97 (-4.65)***	479.81 (23.84)***	-126.47 (-5.87)***	226.28 (17.93)***
Legumes (L)xM				
Extensive	35.68 (8.5)***	143.25 (5.44)***	52.99 (2.01)	122.45 (4.92)***
Less intensive	34.59 (15.31)***	402.99 (7.58)***	68.15 (6.91)***	251.12 (8.31)***
Intensive	28.22 (8.03)***	169.82 (3.1)*	62.41 (3.1)*	107.41 (2.98)*
Highly intensive	32.05 (10.41)***	153.32 (2.85)*	71.68 (3.73)**	115.56 (3.07)*
Very highly intensive	26.87 (13.59)***	54.7 (0.78)	54.94 (3.33)**	42.09 (0.98)
Functional groups (FG)	-16.5 (-6.9)***	-74.73 (-1.94)	-68.82 (-5.46)***	-74.86 (-2.69)*
Grasses (G)	34.08 (8.93)***	167.63 (2.48)	189.36 (10.33)***	170.94 (3.23)**
Tall Herbs (H)	2.92 (0.42)	26.37 (0.51)	17.62 (1.23)	18.4 (0.74)
Random effects {Variance components}				
Block (B)	18.42 {15.43}	<0.0001 {<0.0001}	315.95 {261.11}	106.47 {465.46}
Plot (P)	207.4 {53.25}	12675.99 {4902.78}	1508.65 {131.07}	5140.04 {1952.37}
Residual	195.26 {26.3}	34959.5 {2679.35}	3394.83 {469.45}	13461.4 {1771.81}
Number of groups				
Block	4	4	4	4
Plot	79	79	79	79

Supplementary Table 6: Result of mixed effect model for annual biomass yield (g m^{-2} a^{-1}) and annual revenues (Euro ha^{-1} a^{-1}). *, **, *** denote significance at the 5%, 1% and 0.1% level, respectively (corrected for multiple comparisons). Standard errors are corrected for heteroscedasticity. Numbers in parentheses are z-values, which are based on robust standard errors.

	Biomass yield	Revenues
Constant	10.82 (0.41)	200.7 (1.47)
Plant diversity$^{0.5}$ ($D^{0.5}$)×Management (M)		
Extensive	92.66 (6.5)***	378.36 (5.9)***
Less intensive	100.09 (6.1)***	453.5 (5.69)***
Intensive	128.28 (6.18)***	562.46 (5.34)***
Highly intensive	74.25 (23.3)***	388.47 (9.17)***
Very highly intensive	81.67 (4.08)***	547.66 (4.83)***
M		
Less intensive	148.26 (2.95)**	691.78 (2.99)*
Intensive	343.25 (11.04)***	1776.15 (10.64)***
Highly intensive	318.68 (13.57)***	2011.52 (21.31)***
Very highly intensive	543.28 (25.27)***	3231.98 (66.86)***
Legumes (L)xM		
Extensive	148.33 (5.43)***	893.45 (5.86)***
Less intensive	425.88 (7.33)***	2535.63 (7.25)***
Intensive	174.73 (3.14)**	1356.02 (4.65)***
Highly intensive	155.77 (2.69)**	1396.89 (2.73)*
Very highly intensive	50.6 (0.69)	439.61 (0.96)
Functional groups (FG)	-74.59 (-1.72)	-523.67 (-2.49)
Grasses (G)	167.57 (2.22)*	1064.35 (2.74)*
Tall Herbs (H)	24.82 (0.4)	119.7 (0.41)
Random effects {Variance components}		
Block (B)	<0.0001 {<0.0001}	<0.0001 {<0.0001}
Plot (P)	14603.1 {5673.38}	296129.29 {160445.45}
Residual	41343.77 {3176.59}	1238636.63 {137002.29}
Number of groups		
Block	4	4
Plot	80	79

Differences between Plant Diversity Effects, Model with Legume Presence

Supplementary Table 7: Differences and the corresponding significance levels of the differences between plant diversity effects per management intensity on annual metabolizable energy yield (MJ m^{-2} a^{-1}), annual milk production potential yield (kg m^{-2} a^{-1}) and annual biomass yield (g m^{-2} a^{-1}). 'Management intensity - management intensity' indicates the compared management intensities. *, **, *** denote significance at the 5%, 1% and 0.1% level, respectively (corrected for multiple comparisons). Numbers in parentheses are Wald $\chi 2$ statistics.

Management intensity - management intensity	Metabolizable energy yield	Milk production potential yield	Biomass yield
Extensive - less intensive	-0.13 (0.39)	-0.02 (0.43)	-7.43 (0.1)
Extensive intensive	-0.32 (3.13)	-0.06 (3.32)	-35.63 (2.1)
Extensive - highly intensive	0.02 (0.03)	-0.003 (0.01)	18.41 (1.42)
Extensive - very highly intensive	-0.23 (2.55)	-0.05 (3.89)	10.99 (0.71)
Less intensive - intensive	-0.19 (2.49)	-0.04 (2.3)	-28.19 (5.86)*
Less intensive - highly intensive	0.15 (0.58)	0.02 (0.31)	25.84 (2.82)
Less intensive - very highly intensive	-0.11 (0.32)	-0.03 (0.71)	18.42 (0.93)
Intensive - highly intensive	0.34 (1.98)	0.06 (1.45)	54.03 (8.56)**
Intensive - very highly intensive	0.09 (0.22)	0.005 (0.02)	46.61 (4.75)*
Highly intensive - very highly intensive	-0.26 (1.06)	-0.05 (1.13)	-7.42 (0.13)

Supplementary Table 8: Differences and the corresponding significance levels of the differences between plant diversity effects per management intensity on annual crude protein yield (g m^{-2} a^{-1}), annual organic matter yield (g m^{-2} a^{-1}), annual neutral detergent fiber yield (g m^{-2} a^{-1}) and annual revenues (Euro ha^{-1} a^{-1}). 'Management intensity - management intensity' indicates the compared management intensities. *, **, *** denote significance at the 5%, 1% and 0.1% level, respectively (corrected for multiple comparisons). Numbers in parentheses are Wald χ2 statistics.

Management intensity - management intensity	Crude protein yield	Organic matter yield	Neutral detergent fiber yield	Revenues
Extensive - less intensive	-0.71 (0.07)	-6.28 (0.08)	-0.83 (0.004)	-75.14 (0.43)
Extensive intensive	-5.04 (3.12)	-31.73 (1.95)	-20.42 (3.27)	-184.1 (3.32)
Extensive - highly intensive	-1.55 (0.3)	19.15 (1.57)	35.97 (10.72)**	-10.11 (0.01)
Extensive - very highly intensive	-7.53 (5.43)	12.85 (1.28)	23.83 (31.05)***	-169.31 (3.89)
Less intensive - intensive	-4.32 (1.8)	-25.45 (5.37)	-19.59 (8.45)*	-108.95 (2.3)
Less intensive - highly intensive	-0.84 (0.21)	25.43 (3.28)	36.8 (9.58)*	65.03 (0.31)
Less intensive - very highly intensive	-6.81 (2.5)	19.13 (1.21)	24.66 (4.93)	-94.16 (0.71)
Intensive - highly intensive	3.49 (0.88)	50.88 (9.49)*	56.38 (57.3)***	173.99 (1.45)
Intensive - very highly intensive	-2.49 (0.23)	44.58 (5.19)	44.25 (16.1)***	14.79 (0.02)
Highly intensive - very highly intensive	-5.98 (1.24)	-6.3 (0.11)	-12.14 (1.06)	-159.19 (1.13)

Robustness Analysis – Mixed Effect Model Results, Model with Legume Share

Supplementary Table 9: Robustness analysis (model includes legume shares) – Result of mixed effect model for annual average metabolizable energy content (MJ kg^{-1}), annual metabolizable energy yield (MJ m^{-2} a^{-1}), annual average milk production potential (kg kg^{-1} a^{-1}), annual milk production potential yield (kg m^{-2} a^{-1}). *, **, *** denote significance at the 5%, 1% and 0.1% level, respectively (corrected for multiple comparisons). Standard errors are corrected for heteroscedasticity. Numbers in parentheses are z-values, which are based on robust standard errors.

	Metabolizable energy content	Metabolizable energy yield	Milk production potential	Milk production potential yield
Constant	8.03 (25.78)***	0.06 (0.28)	1.47 (22.58)***	0.01 (0.39)
Plant diversity$^{0.5}$ ($D^{0.5}$)×Management (M)				
Extensive	-0.13 (-1.65)	0.64 (4.58)***	-0.03 (-1.74)	0.11 (4.51)***
Less intensive	-0.07 (-1.54)	0.97 (6.24)***	-0.02 (-1.55)	0.18 (6.22)***
Intensive	-0.11 (-2.65)*	1.01 (5.55)***	-0.03 (-2.63)	0.18 (5.39)***
Highly intensive	0 (0.1)	0.68 (13.79)***	0 (0.05)	0.13 (12.62)***
Very highly intensive	0.05 (1.24)	0.86 (5.01)***	0.01 (1.23)	0.17 (5.13)***
M				
Less intensive	0.59 (1.98)	0.82 (2.17)	0.13 (2.05)	0.15 (2.23)
Intensive	1 (3.67)**	2.86 (19.33)***	0.22 (3.74)**	0.53 (19.54)***
Highly intensive	2.09 (5.51)***	3.22 (32.3)***	0.46 (5.82)***	0.61 (30.65)***
Very highly intensive	2.39 (7.43)***	5.4 (26.45)***	0.53 (7.85)***	1.03 (28.66)***
Legume share ($LS^{0.5}$)xM				
Extensive	0.61 (2.08)	1.01 (2.05)	0.14 (2.15)	0.19 (2.13)
Less intensive	0.95 (3.35)**	5 (15.18)***	0.22 (3.43)**	0.95 (15.17)***
Intensive	0.7 (3.05)*	2.47 (2.84)*	0.16 (3.18)**	0.48 (2.98)*
Highly intensive	0.65 (1.38)	2.36 (1.8)	0.15 (1.37)	0.47 (1.88)
Very highly intensive	0.02 (0.08)	-0.06 (-0.06)	0.01 (0.09)	-0.01 (-0.05)
Functional groups (FG)	-0.03 (-0.25)	-0.39 (-1.15)	-0.01 (-0.33)	-0.08 (-1.24)
Grasses (G)	0.09 (1.55)	1.46 (2.25)	0.03 (1.77)	0.28 (2.36)
Tall Herbs (H)	-0.18 (-0.86)	0.11 (0.19)	-0.04 (-0.84)	0.02 (0.18)
Random effects {Variance components}				
Block (B)	0.004 {0.004}	<0.0001 {<0.0001}	0.0002 {0.0002}	<0.0001 {<0.0001}
Plot (P)	0.08 {0.02}	1.01 {0.42}	0.004 {0.001}	0.03 {0.03}
Residual	0.43 {0.04}	3.63 {0.38}	0.02 {0.002}	0.13 {0.01}
Number of groups				
Block	4	4	4	4
Plot	79	79	79	79

Supplementary Table 10: Robustness analysis (model includes legume shares) – Result of mixed effect model for annual average crude protein content (g kg^{-1}), annual crude protein yield (g m^{-2} a^{-1}), annual average utilizable crude protein content (g kg^{-1}) and annual utilizable crude protein yield (g m^{-2} a^{-1}). *, **, *** denote significance at the 5%, 1% and 0.1% level, respectively (corrected for multiple comparisons). Standard errors are corrected for heteroscedasticity. Numbers in parentheses are z-values, which are based on robust standard errors.

	Crude protein content	Crude protein yield	Utilizable crude protein content	Utilizable crude protein yield
Constant	105.84 (13.79)***	-1.51 (-0.64)	178.31 (24.46)***	7.65 (5.13)***
Plant diversity$^{0.5}$ ($D^{0.5}$)×Management (M)				
Extensive	-4.37 (-5.56)***	8.77 (4.28)***	-	-
Less intensive	-2.49 (-1.16)	14.02 (11.03)***	-3.19 (-1.35)	14.25 (18.8)***
Intensive	-1.92 (-4.02)***	15.47 (4.27)***	-3.1 (-2.31)	12.89 (5.46)***
Highly intensive	-0.65 (-0.37)	11.55 (6.97)***	-0.69 (-0.24)	5.89 (2.58)
Very highly intensive	0.93 (0.42)	16.51 (5.27)***	-0.97 (-0.29)	9.38 (4.26)***
M				
Less intensive	14.48 (1.98)	9.61 (2.1)	-	-
Intensive	18.29 (3.52)**	32.29 (6.32)***	8.53 (1.17)	25.85 (4)***
Highly intensive	75.69 (9.55)***	61.44 (15.28)***	53.85 (7.78)***	4.73 (2.07)
Very highly intensive	98.31 (8.72)***	107.37 (11.57)***	67.12 (4.85)***	13.64 (3.07)*
Legume share ($LS^{0.5}$)xM				
Extensive	86.93 (10.06)***	27.13 (3.16)**	-	-
Less intensive	103.87 (10.42)***	123.16 (18.63)***	28.4 (4.9)***	97.42 (8.46)***
Intensive	79.5 (4.11)***	79.09 (3.36)**	2.41 (0.28)	56.86 (2.3)
Highly intensive	64.34 (8.63)***	59.08 (4.04)***	-3.22 (-0.2)	30.28 (2.94)*
Very highly intensive	54.17 (3.37)**	26.15 (1.06)	-10.16 (-0.8)	26.07 (1.81)
Functional groups (FG)	-8.29 (-1.86)	-8.87 (-1.46)	13.01 (3.41)**	-6.98 (-1.93)
Grasses (G)	-2.32 (-0.39)	19.81 (2.49)	-38.52 (-5.85)***	16.15 (4.3)***
Tall Herbs (H)	8.37 (3.31)**	7.35 (0.6)	-13.34 (-3.15)**	2.24 (0.28)
Random effects {Variance components}				
Block (B)	14.51 {24.17}	<0.0001 {<0.0001}	42.18 {37.41}	19.25 {35.9}
Plot (P)	215.34 {78.69}	361.51 {177.62}	113.92 {38.49}	335.25 {348.56}
Residual	478.08 {52.34}	1108.12 {221.92}	469.55 {91.15}	923.03 {137.42}
Number of groups				
Block	4	4	4	4
Plot	80	80	77	77

Note utilizable crude protein content was only measured for the first cut of the year.

Supplementary Table 11: Robustness analysis (model includes legume shares) – Result of mixed effect model for annual average organic matter content (g kg^{-1}), annual organic matter yield (g m^{-2} a^{-1}), annual average neutral detergent fiber content (g kg^{-1}) and annual neutral detergent fiber yield (g m^{-2} a^{-1}). *, **, *** denote significance at the 5%, 1% and 0.1% level, respectively (corrected for multiple comparisons). Standard errors are corrected for heteroscedasticity. Numbers in parentheses are z-values, which are based on robust standard errors.

	Organic matter content	Organic matter yield	Neutral detergent fiber content	Neutral detergent fiber yield
Constant	888.03 (150.09)***	-12.04 (-0.55)	477.61 (20.23)***	-27.32 (-2.94)*
Plant diversity$^{0.5}$ ($D^{0.5}$)×Management (M)				
Extensive	3.66 (5.87)***	83.57 (5.13)***	24.86 (5.38)***	64.71 (5.41)***
Less intensive	3.4 (3.84)***	108.04 (6.86)***	14.9 (2.95)*	74.62 (5.95)***
Intensive	2.61 (2.71)*	116.34 (6.82)***	19.39 (5.03)***	83.66 (12.13)***
Highly intensive	2.06 (1.06)	66.09 (11.99)***	4.49 (1.09)	28.81 (7.32)***
Very highly intensive	0.81 (2.66)*	67.51 (4.27)***	8.23 (2.33)	37.18 (4.31)***
M				
Less intensive	1.14 (0.17)	94.28 (2.05)	-24.48 (-3.63)**	53.15 (1.7)
Intensive	3.1 (0.46)	291.33 (11.22)***	-27.14 (-1.24)	168.96 (5.46)***
Highly intensive	-15.53 (-2.07)	269.41 (10.51)***	-135.98 (-7.38)***	133.22 (8.87)***
Very highly intensive	-8.88 (-3.11)*	473.66 (16.52)***	-127.16 (-6.16)***	223.98 (14.32)***
Legume share ($LS^{0.5}$)xM				
Extensive	46.58 (9.45)***	95.28 (1.8)	55.71 (1.9)	96.89 (2.64)*
Less intensive	40.25 (11.11)***	471.69 (15.43)***	70.75 (10.17)***	283.94 (18.11)***
Intensive	31.47 (7.22)***	178.48 (1.81)	60.28 (2.34)	102.98 (1.59)
Highly intensive	35.62 (5.42)***	115.13 (1.94)	73.45 (3.37)**	95.97 (2.07)
Very highly intensive	29.37 (6.38)***	-18.23 (-0.2)	61.1 (3.31)**	-5.55 (-0.09)
Functional groups (FG)	-11.52 (-4.12)***	-31.06 (-0.86)	-56.5 (-5.2)***	-44.19 (-1.74)
Grasses (G)	32.51 (8.59)***	136.17 (2.18)	181.17 (12.15)***	146.64 (3.07)*
Tall Herbs (H)	3.35 (0.46)	15.95 (0.28)	15.41 (1.17)	11.8 (0.42)
Random effects {Variance components}				
Block (B)	18.13 {14.88}	<0.0001 {<0.0001}	324.17 {267.41}	196.39 {450.2}
Plot (P)	201.53 {59.03}	13428.88 {5914.07}	1565.97 {218.75}	5445.13 {1727.77}
Residual	193.42 {27.65}	34918.21 {3724.26}	3404.91 {460.79}	13541.5 {1983.81}
Number of groups				
Block	4	4	4	4
Plot	79	79	79	79

Supplementary Table 12: Robustness analysis (model includes legume shares) – Result of mixed effect model for annual biomass yield (g m^{-2} a^{-1}) and annual revenues (Euro ha^{-1} a^{-1}). *, **, *** denote significance at the 5%, 1% and 0.1% level, respectively (corrected for multiple comparisons). Standard errors are corrected for heteroscedasticity. Numbers in parentheses are z-values, which are based on robust standard errors.

	Biomass yield	Revenues
Constant	-11.56 (-0.48)	44.37 (0.39)
Plant diversity$^{0.5}$ ($D^{0.5}$)×Management (M)		
Extensive	90.27 (5.24)***	355.41 (4.51)***
Less intensive	117.07 (6.8)***	546.15 (6.22)***
Intensive	126.82 (6.64)***	567.44 (5.39)***
Highly intensive	73.41 (12.59)***	400.23 (12.62)***
Very highly intensive	75.64 (4.27)***	514.24 (5.13)***
M		
Less intensive	109.49 (2.29)*	464.1 (2.23)
Intensive	325.27 (12.65)***	1646.41 (19.54)***
Highly intensive	311.24 (11.52)***	1895.4 (30.65)***
Very highly intensive	537.36 (17.76)***	3195.64 (28.66)***
Legume share ($\mathrm{LS}^{0.5}$)xM		
Extensive	94.88 (1.7)	600.86 (2.13)
Less intensive	495.88 (16.77)***	2955.23 (15.17)***
Intensive	180.23 (1.77)	1496.89 (2.98)*
Highly intensive	110.42 (1.76)	1441.59 (1.88)
Very highly intensive	-31.51 (-0.35)	-27.85 (-0.05)
Functional groups (FG)	-28.03 (-0.69)	-235.3 (-1.24)
Grasses (G)	132.59 (1.9)	871.45 (2.36)
Tall Herbs (H)	12.44 (0.18)	59.64 (0.18)
Random effects {Variance components}		
Block (B)	<0.0001 {<0.0001}	<0.0001 {0.001}
Plot (P)	15488.5 {13392.26}	328136.2 {135751.07}
Residual	41314.4 {4062.21}	1233574.1 {143544.79}
Number of groups		
Block	4	4
Plot	80	79

Differences between Plant Diversity Effects, Model with Legume Presence

Supplementary Table 13: Robustness analysis (model includes legume shares) – Differences and the corresponding significance levels of the differences between plant diversity effects per management intensity on annual metabolizable energy yield (MJ m^{-2} a^{-1}), annual milk production potential yield (kg m^{-2} a^{-1}) and annual biomass yield (g m^{-2} a^{-1}). 'Management intensity - management intensity'indicates the compared management intensities. *, **, *** denote significance at the 5%, 1% and 0.1% level, respectively (corrected for multiple comparisons). Numbers in parentheses are Wald χ2 statistics.

Management intensity - management intensity	Metabolizable energy yield	Milk production potential yield	Biomass yield
Extensive - less intensive	-0.32 (2.79)	-0.06 (3.15)	-26.8 (1.24)
Extensive intensive	-0.36 (3.7)	-0.07 (4.02)	-36.55 (2.07)
Extensive - highly intensive	-0.03 (0.03)	-0.014 (0.2)	16.86 (0.83)
Extensive - very highly intensive	-0.21 (4.1)	-0.05 (6.86)	14.62 (1.74)
Less intensive - intensive	-0.04 (0.61)	-0.01 (0.45)	-9.75 (6.12)*
Less intensive - highly intensive	0.29 (2.27)	0.05 (1.66)	43.66 (11.29)***
Less intensive - very highly intensive	0.11 (0.46)	0.01 (0.12)	41.42 (6.28)*
Intensive - highly intensive	0.33 (2.29)	0.05 (1.65)	53.41 (14.44)***
Intensive - very highly intensive	0.15 (0.81)	0.017 (0.31)	51.17 (7.53)**
Highly intensive - very highly intensive	-0.18 (0.69)	-0.04 (0.76)	-2.24 (0.02)

Supplementary Table 14: Differences and the corresponding significance levels of the differences between plant diversity effects per management intensity on annual crude protein yield (g m^{-2} a^{-1}), annual organic matter yield (g m^{-2} a^{-1}), annual neutral detergent fiber yield (g m^{-2} a^{-1}) and annual revenues (Euro ha^{-1} a^{-1}). 'Management intensity - management intensity' indicates the compared management intensities. *, **, *** denote significance at the 5%, 1% and 0.1% level, respectively (corrected for multiple comparisons). Numbers in parentheses are Wald $\chi 2$ statistics.

Management intensity - management intensity	Crude protein yield	Organic matter yield	Neutral detergent fiber yield	Revenues
Extensive - less intensive	-5.25 (6.1)	-24.47 (1.19)	-9.92 (0.486)	-190.75 (3.15)
Extensive intensive	-6.7 (4.13)	-32.77 (1.93)	-18.95 (2.26)	-212.04 (4.02)
Extensive - highly intensive	-2.78 (0.71)	17.48 (0.91)	35.9 (8.3)*	-44.83 (0.2)
Extensive - very highly intensive	-7.74 (12.36)**	16.06 (2.68)	27.53 (31.03)***	-158.83 (6.86)
Less intensive - intensive	-1.45 (0.33)	-8.3 (4.6)	-9.04 (1.87)	-21.29 (0.45)
Less intensive - highly intensive	2.47 (2.71)	41.95 (12.94)**	45.82 (18.95)***	145.92 (1.66)
Less intensive - very highly intensive	-2.49 (0.51)	40.53 (7.24)*	37.45 (11.97)**	31.91 (0.12)
Intensive - highly intensive	3.92 (1.5)	50.25 (16.96)***	54.85 (187.53)***	167.21 (1.65)
Intensive - very highly intensive	-1.04 (0.04)	48.83 (8.17)*	46.48 (25.27)***	53.2 (0.31)
Highly intensive - very highly intensive	-4.97 (1.12)	-1.42 (0.01)	-8.37 (0.77)	-114.01 (0.76)

Supplementary Table 15: Pearson correlation coefficient of annual biomass yield (g m^{-2} a^{-1}) and annual metabolizable energy yield (MJ m^{-2} a^{-1}), annual milk production potential yield (kg m^{-2} a^{-1}), annual crude protein yield (g m^{-2} a^{-1}), annual utilizable crude protein yield (g m^{-2} a^{-1}), annual organic matter yield (g m^{-2} a^{-1}) and annual neutral detergent fiber yield (g m^{-2} a^{-1}). Note utilizable crude protein content was only measured for the first cut of the year, therefore, the Pearson correlation coefficient refers to biomass yield and utilizable crude protein yield of the first cut.

	Biomass yield
Metabolizable energy yield	0.98
Milk production potential yield	0.97
Crude protein yield	0.88
Utilizable crude protein yield	0.96
Organic matter yield	1.00

Supplementary Table 16: Cutting dates in 2007. Biomass yield samples of dates given in italics were only quantified and not analyzed for forage quality variables. See Supplementary Table 1 for details on management intensities.

Management intensity	Cutting season			
	Spring			Autumn
Extensive	--	--	--	September 1
Less intensive	June 4	--	--	September 1
Intensive	June 4	--	--	September 1
Highly intensive	April 24	*June 4*	*July 23*	September 1
Very highly intensive	April 24	*June 4*	*July 23*	September 1

Supplementary Figures

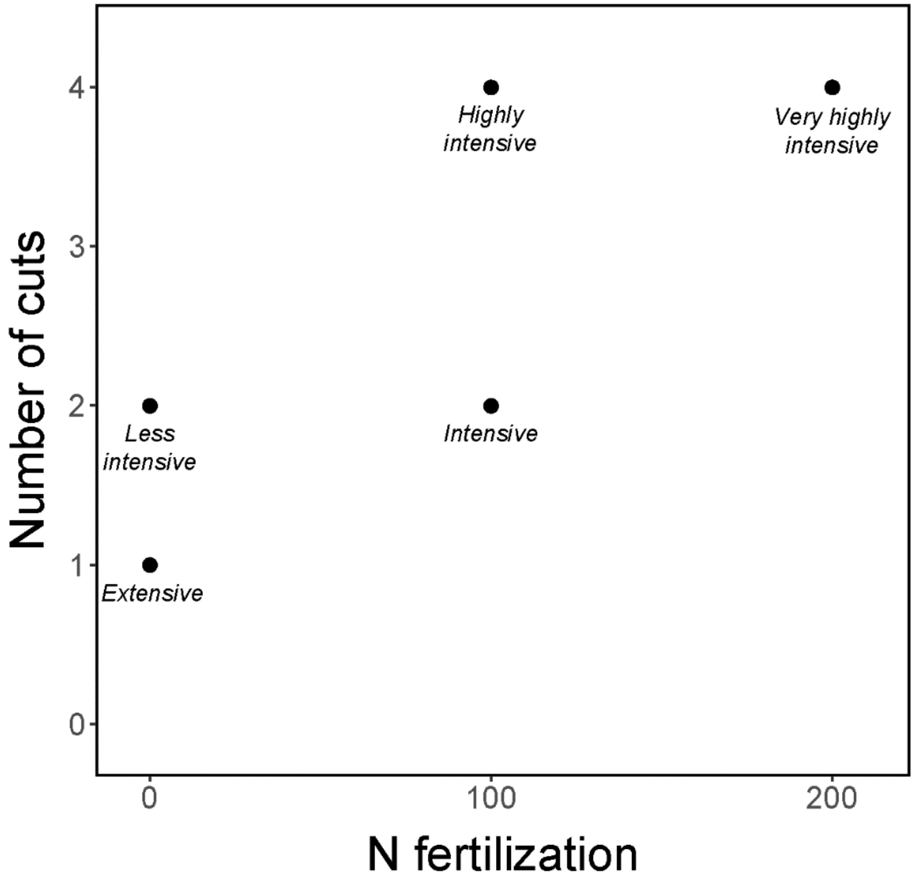

Supplementary Figure 1: Illustration of the management intensities and their elements (cutting frequency and N fertilization (kg ha^{-1} a^{-1}) per year) tested in our study. The experimental design includes five different management intensities, which are here defined as: extensive, less intensive, intensive, highly intensive and very highly intensive management. Beside N, the fertilization included P and K (see Supplementary Table 2).

Supplementary References

1. Ball, D. M. et al. *Understanding Forage Quality* (American Farm Bureau Federation Publication, Park Ridge, 2001).

2. Barnes, R. F., Nelson, C. J., Collins, M. & Moore, K. J. *Forages. Volume 1: An Introduction to Grassland Agriculture*. (Iowa State Press, Iowa, 2003).

3. Edmunds, B., Südekum, K.-H., Spiekers, H., Schuster, M. & Schwarz, F. J. Estimating utilisable crude protein at the duodenum, a precursor to metabolisable protein for ruminants, from forages using a modified gas test. *Anim. Feed Sci. Technol.* **175**, 106-113 (2012).

4. Jans, F., Kessler, J., Münger, A. & Schlegel, P. in *Fütterungsempfehlungen für Wiederkäuer (Grünes Buch)* Ch. 7 (Agroscope, Posieux, 2015).
