# Supplementary material for: Plant diversity effects on forage quality, yield and revenues of semi-natural grasslands
Source: Nat Commun. 2020 Feb 7;11:768. doi: 10.1038/s41467-020-14541-4 (PMC7005841; doi:10.1038/s41467-020-14541-4)
Supplement: Supplementary file 3 — Description of Additional Supplementary Information [file 41467_2020_14541_MOESM3_ESM.docx]

**Description of Additional Supplementary Files**

**File Name:** Supplementary Code Files 1
**Description:** Code for Data Processing

**File Name:** Supplementary Code Files 2
**Description:** Code for Main Analysis

**File Name:** Supplementary Code Files 3
**Description:** Code for Robustness Analysis
